# Stark Many-Body Localization-Induced Quantum Mpemba Effect

**DOI:** 10.3390/e28030348

**Published:** 2026-03-19

**Authors:** Yi-Rui Zhang, Han-Ze Li, Xu-Yang Huang, Yu-Jun Zhao, Jian-Xin Zhong

**Affiliations:** 1 Institute for Quantum Science and Technology, Shanghai University, Shanghai 200444, Chinahanzeli@u.nus.edu (H.-Z.L.);; 2 Department of Physics, National University of Singapore, Singapore 117542, Singapore; 3 School of Physics and Optoelectronics, Xiangtan University, Xiangtan 411105, China

**Keywords:** stark many-body localization, quantum mpemba effect, entanglement asymmetry, symmetry restoration, non-equilibrium dynamics

## Abstract

The quantum Mpemba effect (QME) describes the counterintuitive phenomenon where a system initially further from equilibrium relaxes faster than one closer to it. Specifically, the QME associated with symmetry restoration has been extensively investigated across integrable, ergodic, and disordered localized systems. However, its fate in disorder-free ergodicity-breaking settings, such as the Stark many-body localized (Stark-MBL) phase, remains an open question. Here, we explore the dynamics of local U(1) symmetry restoration in a Stark-MBL XXZ spin-$\frac{1}{2}$ chain, using the Rényi-2 entanglement asymmetry (EA) as a probe. Using an analytical operator-string expansion supported by numerical simulations, we demonstrate that the QME transitions from an initial-state-dependent anomaly in the ergodic phase to a universal feature in the Stark-MBL regime. Moreover, the Mpemba time scales exponentially with the subsystem size, even in the absence of global transport, and is governed by high-order off-resonant processes. We attribute this robust inversion to a Stark-induced hierarchy of relaxation channels that fundamentally constrains the effective Hilbert space dimension. The findings pave the way for utilizing tunable potentials to engineer and control anomalous relaxation timescales in quantum technologies without reliance on quenched disorder.

## 1. Introduction

Understanding how isolated quantum many-body systems lose locally accessible information about their initial conditions is a central problem in non-equilibrium physics [1,2]. In generic interacting settings, the eigenstate thermalization hypothesis [3,4] suggests that after a global quench, small subsystems approach stationary reduced states determined by conservation laws. When a global U(1) charge *Q* is conserved, this relaxation has a particularly transparent structural signature: the reduced density matrix of a subregion, $\rho_{A}\left(t\right)$, progressively becomes compatible with the charge-sector decomposition set by the restricted charge $Q_{A}$, so that coherences between different $Q_{A}$ sectors are suppressed and local properties become consistent with an equilibrium description constrained by energy and charge [5,6].

This structural relaxation is commonly referred to as symmetry restoration [7,8,9,10,11,12,13,14,15] at the subsystem level. To diagnose this, the EA [16] is utilized: a symmetry-sensitive quantity constructed from $\rho_{A}\left(t\right)$ that measures the remaining locally accessible symmetry-breaking information and vanishes when $\rho_{A}\left(t\right)$ becomes symmetric with respect to $Q_{A}$. Because it is defined solely in terms of the reduced state and directly targets inter-sector coherence, EA provides an experimental and theoretical proof of symmetry restoration in unitary dynamics. Symmetry restoration also exposes a counterintuitive ordering phenomenon. The QME (see recent reviews [17,18]) captures the possibility that, under identical dynamics, an initial state that is farther from the stationary structure can relax faster than one that starts closer. Quantum Mpemba behaviour has been explored in both open [19,20,21,22,23,24,25,26,27,28,29,30,31,32,33,34,35,36,37,38,39,40,41,42,43] and closed [16,44,45,46,47,48,49,50,51,52,53,54,55,56,57,58,59,60,61,62,63,64,65] systems. Here, unless stated otherwise, we use the term QME exclusively in the closed-system sense, where the effect is diagnosed through local equilibration under unitary time evolution. Recent works [16,44,45,46,47,48,49,50,51,52,53,54,55,56,57,58,59,60,61,62,63,64,65] have shown that this closed-system Mpemba phenomenology arises in Hamiltonian many-body dynamics and in symmetric random quantum circuits, where conservation laws are enforced at the level of local gates and the dynamics provides a minimal setting for unitary thermalization. In the symmetry-restoration context, the Mpemba effect corresponds to an inversion of restoration times, meaning that a more strongly symmetry-broken initial condition can restore symmetry faster according to symmetry-resolved diagnostics such as EA.

Thus, the key motivation of this work is to understand whether such symmetry-restoration Mpemba inversions persist when ergodicity is impeded. Localization [66] provides a natural route to non-ergodic unitary dynamics and long-lived memory, and it reshapes relaxation by restricting which parts of Hilbert space are dynamically explored on accessible time scales. This makes symmetry restoration strongly preparation-dependent and suggests a natural mechanism for inversions [58]: different initial states can expand the effectively explored, symmetry-allowed Hilbert space at different rates, leading to different rates of sector redistribution and intra-sector mixing in $\rho_{A}\left(t\right)$, and hence to different decay rates of EA.

Many-body localization has already provided evidence that Mpemba-type inversions can occur even in non-ergodic dynamics [58]. This raises an immediate and practically important question: Is the phenomenon tied to quenched randomness, or does it persist in non-ergodic settings driven by a different microscopic mechanism? Stark-MBL [67,68,69] offers a complementary route to localization that is induced by a uniform potential gradient rather than disorder. It avoids sample-to-sample fluctuations, reduces the reliance on disorder averaging, and introduces a continuously tunable control parameter, the field strength, that allows one to traverse ergodic and non-ergodic regimes within a single Hamiltonian family. These features make Stark-MBL an ideal platform for interrogating symmetry restoration and its Mpemba inversion in a controlled manner, and for connecting the inversion directly to how a potential gradient reorganizes resonant processes and constrains the growth of the dynamically explored Hilbert space.

**Figure 1:**
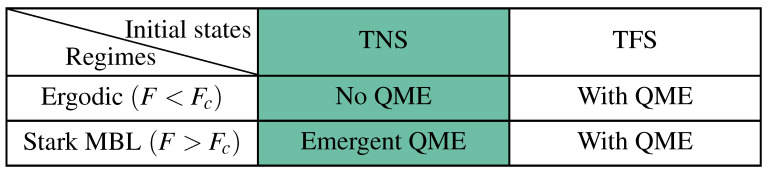
Comparison of the presence of QME in the ergodic region (first row) and the Stark-MBL region (second row) for the different initial states (TFS and TNS) considered in this work.

In this work, we provide a comprehensive analysis of symmetry restoration and the QME in the context of Stark-MBL. By investigating the dynamics of the Rényi-2 EA in a tilted XXZ chain, we found that the Stark-induced breaking of ergodicity gives rise to a robust and universal Mpemba effect. Our central findings regarding the phase dependence of the QME are summarized below (see the table in Figure 1):

- In the ergodic phase, the occurrence of the QME is contingent upon the microscopic details of the initial state. Specifically, it is present for the Tilted Ferromagnetic State (TFS) but absent for the Tilted Néel State (TNS), as the relaxation is governed by the dimension of the dynamically accessible charge sectors.
- In the Stark-MBL phase, the QME becomes a universal phenomenon. It emerges for both TNS and TFS initial states. This universality arises from a Stark-induced inversion of the steady-state ordering, where stronger initial symmetry breaking leads to a more symmetric local steady state due to the restricted effective Hilbert space dimension.

Furthermore, we analytically determined the characteristic timescale of this anomalous relaxation. We found that the Mpemba time $t_{M}$ is governed by high-order perturbative processes and scales exponentially with the subsystem size, $t_{M}\propto {\left(F/J\right)}^{N_{A}-1}$. This prediction is quantitatively corroborated by exact diagonalization simulations, confirming that symmetry restoration in the Stark-MBL phase is driven by slow, off-resonant quantum fluctuations rather than diffusive transport.

These results place Mpemba-type anomalies in a tunable localization setting without quenched disorder, providing a clear protocol for observing this effect in state-of-the-art quantum simulators such as trapped ions or superconducting circuits. Broadly, our work paves the way for utilizing tunable potentials to engineer anomalous relaxation timescales, providing new insights into the interplay between ergodicity breaking and symmetry-protected quantum information.

## 2. Methods

### 2.1. Model Hamiltonian

We study a one-dimensional spin-1/2 XXZ chain in the presence of a linear Stark potential. The Hamiltonian is given by:

(1) $$H=-J\sum_{i=1}^{N-1}\left(S_{i}^{x}S_{i+1}^{x}+S_{i}^{y}S_{i+1}^{y}\right)+\Delta \sum_{i=1}^{N-1}S_{i}^{z}S_{i+1}^{z}+\sum_{i=1}^{N}\left[Fi+{\left(i/N\right)}^{2}\right]S_{i}^{z},$$

where $S_{i}^{\alpha }$ (α∈{x,y,z}) are the spin-1/2 operators at site *i*, satisfying the commutation relations $\left[S_{i}^{\alpha },S_{j}^{\beta }\right]=i\delta_{ij}\epsilon_{\alpha \beta \gamma }S_{i}^{\gamma }$. Here, *J* sets the energy scale of the hopping set to J=2 thereafter; Δ represents the nearest-neighbor interaction anisotropy; and *F* denotes the strength of the effective electric field, the Stark potential gradient. We employ open boundary conditions to accommodate the linear potential. The quadratic term ${\left(i/N\right)}^{2}$ is included as a smooth boundary regularization to gently lift finite-size near-degeneracies of the perfectly linear tilt and suppress long-lived coherent oscillations, thereby stabilizing the late-time dephasing behavior [67].

The model conserves the total magnetization $Q\;=\;\sum_{i}S_{i}^{z}$, exhibiting a global U(1) symmetry. The interplay between the interaction Δ and the tilt *F* governs the transition from an ergodic thermalizing phase to a Stark-MBL phase, in which transport is suppressed, and the system breaks ergodicity.

### 2.2. R Ényi-2 Entanglement Asymmetry

To quantify the restoration of the U(1) symmetry within a local subsystem, we utilize the EA. We partition the system into a subsystem *A* of size $N_{A}$ and its complement *B*. The state of the subsystem is described by the reduced density matrix $\rho_{A}\left(t\right)={\mathrm{Tr}}_{B}\left[|\psi (t)\rangle \langle \psi (t)|\right]$. The global U(1) symmetry implies the existence of a local charge operator $Q_{A}=\sum_{i\in A}S_{i}^{z}$. If the symmetry is locally broken, $[\rho_{A}\left(t\right),Q_{A}]\neq 0$. The symmetrized state, which retains only the diagonal blocks in the charge basis, is obtained via the twirling map:

(2) $$\rho_{A,Q}\left(t\right)=\sum_{q}\Pi_{q}\rho_{A}\left(t\right)\Pi_{q}=\frac{1}{2\pi }\int_{0}^{2\pi }d\phi \;e^{-i\phi Q_{A}}\rho_{A}\left(t\right)e^{i\phi Q_{A}},$$

where $\Pi_{q}$ is the projector onto the eigenspace of $Q_{A}$ with charge *q*. The EA is defined as the difference between the entanglement entropy of the symmetrized state and the original state. We specifically employ the Rényi-2 EA, $\Delta S_{A}^{\left(2\right)}$, due to its analytical tractability:

(3) $$\Delta S_{A}^{\left(2\right)}\left(t\right)=S^{\left(2\right)}\left(\rho_{A,Q}\left(t\right)\right)-S^{\left(2\right)}\left(\rho_{A}\left(t\right)\right),$$

where the Rényi-2 entropy is $S^{\left(2\right)}\left(\rho \right)=-\log\mathrm{Tr}\left(\rho^{2}\right)$. Substituting this definition, the asymmetry can be expressed as the logarithm of the ratio of purities:

(4) $$\Delta S_{A}^{\left(2\right)}\left(t\right)=\log\left(\frac{\mathrm{Tr}(\rho_{A}^{2}\left(t\right))}{\mathrm{Tr}(\rho_{A,Q}^{2}\left(t\right))}\right).$$

This quantity is strictly non-negative, $\Delta S_{A}^{\left(2\right)}\left(t\right)\geq 0$, and vanishes if and only if the state is U(1) symmetric, i.e., $[\rho_{A},Q_{A}]=0$.

### 2.3. Operator Space Expansion

To analyze the dynamical mechanism of symmetry restoration, it is convenient to expand the density matrix in an orthonormal operator basis. The set of operator strings $P=\prod_{i\in A}O_{i}$, where $O_{i}\in \left\{I_{i},S_{i}^{+},S_{i}^{-},S_{i}^{z}\right\}$, forms a complete basis for operators on subsystem *A* with $S_{i}^{\pm }\;=\;\frac{1}{\sqrt{2}}\left(S_{i}^{x}\pm iS_{i}^{y}\right)$. We normalize the basis such that $\mathrm{Tr}\left(P^{†}P^{\prime }\right)=2^{N_{A}}\delta_{P,P^{\prime }}$. The purity of the reduced density matrix can then be expanded as follows:

(5) $$\mathrm{Tr}\left(\rho_{A}^{2}\left(t\right)\right)=\frac{1}{2^{N_{A}}}\sum_{P\subset A}{\left|\langle \psi (t)|P|\psi (t)\rangle \right|}^{2}\equiv \frac{1}{2^{N_{A}}}f\left(t\right),$$

where f(t) represents the total weight of all operator strings.

Similarly, the purity of the symmetrized state $\rho_{A,Q}$ contains contributions only from charge-conserving strings, those satisfying $[P,Q_{A}]=0$:

(6) $$\mathrm{Tr}\left(\rho_{A,Q}^{2}\left(t\right)\right)=\frac{1}{2^{N_{A}}}\sum_{P\subset A:[P,Q_{A}]=0}{\left|\langle \psi (t)|P|\psi (t)\rangle \right|}^{2}\equiv \frac{1}{2^{N_{A}}}f^{c}\left(t\right).$$

Substituting these into Equation (4), the Rényi-2 EA, then, becomes

(7) $$\Delta S_{A}^{\left(2\right)}\left(t\right)=\log\left(\frac{f(t)}{f^{c}\left(t\right)}\right).$$

Here, f(t) sums over all strings, while $f^{c}\left(t\right)$ sums only over the diagonal strings that preserve the local charge.

The dynamics of $\Delta S_{A}^{\left(2\right)}\left(t\right)$, Equation (7), are thus governed by the relative decay rates of charge-neutral versus charge-non-conserving operator strings.

### 2.4. Quantum Mpemba Effect

The QME describes an anomalous relaxation phenomenon in which a system that is initially farther from equilibrium relaxes faster than one that is initially closer. We consider a family of initial states parametrized by a tilt angle θ, denoted as $|\psi_{\theta }\rangle$. The distance from symmetry at time *t* is quantified by $\Delta S_{A}^{\left(2\right)}\left(t;\theta \right)$. We assume that the initial asymmetry $\Delta S_{A}^{\left(2\right)}\left(0;\theta \right)$ is monotonically increasing with $\theta \;\in \;(0,\frac{\pi }{2}]$, i.e., “hotter” states have larger initial asymmetry.

A QME is defined to occur iff there exist two parameters $\theta_{h}>\theta_{c}$ such that

(8) $$\Delta S_{A}^{\left(2\right)}\left(0;\theta_{h}\right)>\Delta S_{A}^{\left(2\right)}\left(0;\theta_{c}\right),$$

but at some later time $t>t_{M}$ (the Mpemba time):

(9) $$\Delta S_{A}^{\left(2\right)}\left(t;\theta_{h}\right)<\Delta S_{A}^{\left(2\right)}\left(t;\theta_{c}\right).$$

Specifically, we investigate the existence of this in the Stark-MBL regime, where non-ergodicity may protect certain operator sectors, leading to non-trivial relaxation hierarchies.

## 3. Results

### 3.1. Analytical Results

#### 3.1.1. From the Stark Hamiltonian to an Effective Diagonal Description

We start from Equation (1) and focus on the strong-tilt regime F≫J,Δ. We decompose $H=H_{0}+V$ with

(10) $$\begin{array}{cc} H_{0} & =\sum_{i=1}^{N}\left[Fi+{\left(i/N\right)}^{2}\right]S_{i}^{z}+\Delta \sum_{i=1}^{N-1}S_{i}^{z}S_{i+1}^{z}, \end{array}$$

(11) $$\begin{array}{cc} V & =-J\sum_{i=1}^{N-1}\left(S_{i}^{x}S_{i+1}^{x}+S_{i}^{y}S_{i+1}^{y}\right)=-\frac{J}{2}\sum_{i=1}^{N-1}\left(S_{i}^{+}S_{i+1}^{-}+S_{i}^{-}S_{i+1}^{+}\right). \end{array}$$

In the Dirac picture with respect to $H_{0}$, each nearest-neighbor flip-flop term acquires a rapidly oscillating phase $e^{\pm i[\left(\varepsilon_{i+1}-\varepsilon_{i}\right)+\Delta \;\delta_{i}]t}$, where $\varepsilon_{i}=Fi+{\left(i/N\right)}^{2}$ and $\delta_{i}$ is an O(1) operator-valued shift set by the adjacent $S^{z}$ configuration. Since $\varepsilon_{i+1}-\varepsilon_{i}≃F$ dominates for F≫J,Δ, these terms are strongly off-resonant and average out on the long-time scales relevant for the late-time envelopes discussed below.

Consequently, to leading order in J/F, the dynamics is governed by an effective Hamiltonian obtained by perturbatively eliminating *V* (equivalently via a Schrieffer–Wolff transformation). In the strong-Stark limit this effective Hamiltonian is (quasi-)diagonal in the computational basis and takes the generic form

(12) $$H_{\mathrm{eff}}=\sum_{i}h_{i}S_{i}^{z}+\sum_{i<j}J_{ij}S_{i}^{z}S_{j}^{z}+\cdots ,$$

where $h_{i}\approx Fi+{\left(i/N\right)}^{2}$ up to $O(J^{2}/F)$ corrections, and the longitudinal couplings $J_{ij}$ arise from virtual sequences of the flip-flop processes and decay rapidly with distance. For our purposes, the crucial point is that $H_{\mathrm{eff}}$ generates configuration-dependent phases in the $S^{z}$ basis, which dephase expectation values of operator strings containing $S^{\pm }$ at late times.

#### 3.1.2. Stark Many-Body Localized EA Plateau

We now derive the late-time plateau rules for the operator-string weights entering the Rényi-2 EA (7). We consider the same tilted product initial-state families as in the main text, namely the TFS and the TNS. Let {|0〉,|1〉} denote the local eigenstates of the computational basis $S_{i}^{z}$. The TFS is the uniform tilted product state

(13) $$|\psi_{\theta }^{\mathrm{TFS}}\rangle ={\left(\cos\left(\frac{\theta }{2}\right)|0\rangle +\sin\left(\frac{\theta }{2}\right)|1\rangle \right)}^{⊗N}.$$

The TNS differs only by a staggered relative phase between the |0〉 and |1〉 components,

(14) $$|\psi_{\theta }^{\mathrm{TNS}}\rangle =\overset{N}{\underset{i=1}{⨂}}(\cos\left(\frac{\theta }{2}\right)|0\rangle +{\left(-1\right)}^{i}\sin\left(\frac{\theta }{2}\right)|1\rangle ).$$

In both cases, the computational-basis probabilities are identical and site-independent, $|\langle \sigma |\psi_{\theta }\rangle {|}^{2}=\prod_{i=1}^{N}p_{\sigma_{i}}$ with $p_{0}=\cos^{2}\left(\theta /2\right)$ and $p_{1}=\sin^{2}\left(\theta /2\right)$.

In the effective diagonal dynamics of Section 3.1.1, operator strings containing at least one $S^{\pm }$ acquire rapidly varying configuration-dependent phases. After long-time averaging, off-diagonal contributions dephase, yielding a diagonal-probability expression whose magnitude factorizes into single-site contributions. Each site on which *P* contains $S^{\pm }$ enforces a flip between the paired computational configurations and contributes a factor $2p_{0}p_{1}$, whereas each site with *I* or $S^{z}$ contributes $p_{0}^{2}+p_{1}^{2}$. It is therefore convenient to define

(15) $$\begin{array}{cc} a_{\theta } & \equiv p_{0}^{2}+p_{1}^{2}=\cos^{4}\left(\theta /2\right)+\sin^{4}\left(\theta /2\right)=\frac{1+\cos^{2}\theta }{2}, \end{array}$$

(16) $$\begin{array}{cc} \;b_{\theta } & \equiv 2p_{0}p_{1}=2\cos^{2}\left(\theta /2\right)\sin^{2}\left(\theta /2\right)=\frac{\sin^{2}\theta }{2}. \end{array}$$

Then for any operator string with $n_{\mathrm{chg}}\left(P\right)=\#\left(S^{+}\right)+\#\left(S^{-}\right)\geq 1$, the late-time plateau obeys

(17) $$\bar{{\left|\langle \psi (t)|P|\psi (t)\rangle \right|}^{2}}≃b_{\theta }^{\;n_{\mathrm{chg}}\left(P\right)}\;a_{\theta }^{\;N-n_{\mathrm{chg}}\left(P\right)}.$$

The factor $a_{\theta }^{\;N-n_{\mathrm{chg}}\left(P\right)}$ includes contributions from sites outside *A* because the long-time dephasing expression involves full-system probabilities $|\langle \sigma |\psi_{\theta }\rangle {|}^{4}$. For purely diagonal strings $P\in {\left\{I,S^{z}\right\}}^{⊗N_{A}}$, one has $[P,H_{\mathrm{eff}}]=0$, so their expectation values are time independent. Summing their squared expectations in the above operator normalization gives the exact identity

(18) $$\sum_{P\in {\left\{I,S^{z}\right\}}^{⊗N_{A}}}{\left|\langle \psi (t)|P|\psi (t)\rangle \right|}^{2}={\left(1+\cos^{2}\theta \right)}^{N_{A}}=2^{N_{A}}a_{\theta }^{N_{A}}.$$

We now compute the long-time limits of f(t) and $f^{c}\left(t\right)$ defined in Section 2. A convenient way to obtain $f\left(\theta ,\infty \right)=\sum_{P\subset A}\bar{{\left|\langle P\left(t\right)\rangle \right|}^{2}}$ is to (i) apply the plateau rule Equation (17) to all $4^{N_{A}}$ strings and (ii) to replace the purely diagonal subset $P\in {\left\{I,S^{z}\right\}}^{⊗N_{A}}$ by its exact contribution Equation (18).

Step (i): Summing Equation (17) over all strings amounts to summing, on each site in *A*, the four local contributions *I*, $S^{z}$, $S^{+}$, $S^{-}$, which yields

(19) $$\left(a_{\theta }+a_{\theta }+b_{\theta }+b_{\theta }\right)=2\left(a_{\theta }+b_{\theta }\right)=2,$$

since $a_{\theta }+b_{\theta }={\left(p_{0}+p_{1}\right)}^{2}=1$. Thus, the plateau sum over all strings is $2^{N_{A}}a_{\theta }^{\;N-N_{A}}$.

Step (ii): Within that plateau sum, the diagonal subset contributes $2^{N_{A}}a_{\theta }^{\;N}$, while its exact value is $2^{N_{A}}a_{\theta }^{\;N_{A}}$. Replacing the diagonal subset, therefore, gives

(20) $$f\left(\theta ,\infty \right)=2^{N_{A}}\left(a_{\theta }^{N_{A}}+a_{\theta }^{N-N_{A}}-a_{\theta }^{N}\right).$$

For $f^{c}\left(\theta ,\infty \right)$, by definition, only charge-neutral strings with $[P,Q_{A}]=0$ contribute. All diagonal strings are charge-neutral, hence $f^{c}\left(\theta ,\infty \right)\geq 2^{N_{A}}a_{\theta }^{N_{A}}$. Additional charge-neutral contributions arise from strings containing equal numbers of $S^{+}$ and $S^{-}$ within *A*. These form a strict subset of the spin-changing strings, and their total contribution is bounded by the total spin-changing weight, giving

(21) $$0\leq f^{c}\left(\theta ,\infty \right)-2^{N_{A}}a_{\theta }^{N_{A}}\leq 2^{N_{A}}\left(a_{\theta }^{N-N_{A}}-a_{\theta }^{N}\right).$$

In finite *N* with $\theta \in (0,\frac{\pi }{2}]$ so that $a_{\theta }<1$ and $N_{A}/N<1/2$, this correction is parametrically small compared with $2^{N_{A}}a_{\theta }^{N_{A}}$. To leading order we therefore take

(22) $$f^{c}\left(\theta ,\infty \right)≃2^{N_{A}}a_{\theta }^{N_{A}}.$$

Thus, the steady-state plateau prediction $\Delta S_{A}^{\left(2\right)}\left(\infty \right)=\log\frac{f(\theta ,\infty )}{f^{c}\left(\theta ,\infty \right)}≃\log\;\left[1+a_{\theta }^{\;N-2N_{A}}-a_{\theta }^{\;N-N_{A}}\right]$. When $a_{\theta }^{N-2N_{A}}\ll 1$, we obtain

(23) $$\begin{array}{c} \Delta S_{A}^{\left(2\right)}\left(\infty \right)≃a_{\theta }^{N-2N_{A}}\left(1-a_{\theta }^{N_{A}}\right). \end{array}$$

Note that the above plateau rule depends on the initial state only through the computational-basis probabilities $|\langle \sigma |\psi_{\theta }\rangle {|}^{2}$. Since TFS and TNS differ only by site-dependent phases, they share the same $|\langle \sigma |\psi_{\theta }\rangle {|}^{2}$ and hence yield identical plateau predictions for f(∞), $f^{c}\left(\infty \right)$, and $\Delta S_{A}^{\left(2\right)}\left(\infty \right)$.

#### 3.1.3. Emergent QME in Stark Many-Body Localization and Mpemba-Time Scaling

We now elucidate the physical mechanism underlying the emergent QME and its Rényi-2 EA QME time scaling properties.

First, we address the question of symmetry restoration. In the Stark-MBL regime, the system is non-ergodic and does not relax to a thermal Gibbs ensemble. However, this does not imply that local subsystems retain all information about the initial symmetry breaking. As shown by Equations (20) and (22), in the thermodynamic limit N→∞ with fixed subsystem size $N_{A}$, the steady-state EA is controlled by the term $a_{\theta }^{N-2N_{A}}$. Since $a_{\theta }<1$ for any θ>0, this term vanishes as N→∞. Consequently, $\Delta S_{A}^{\left(2\right)}\left(\infty \right)\to 0$. This indicates that even in the absence of global transport, the dephasing induced by the Stark potential is sufficient to destroy off-diagonal coherences between charge sectors locally, thereby restoring the U(1) symmetry at the subsystem level.

Second, we determine whether the QME exists and its physical origin. The Stark-MBL regime is governed by a fundamentally different mechanism than the ergodic regime, leading to distinct behaviors for the TNS [also see Figure 1]:

- Ergodic Regime: Symmetry restoration is governed by the dimension of the dynamically accessible Hilbert space sectors.
  – For the TNS, the untilted state (θ=0) resides in the half-filling sector (Q=0), which has the maximal Hilbert space dimension and thus the fastest thermalization rate. Increasing the tilt θ spreads the wavefunction into charge sectors away from half-filling, which have smaller dimensions and slower relaxation rates. Since the state with larger initial asymmetry relaxes slower, the EA curves do not cross, and QME is absent for the TNS.
  – For the TFS, the situation is reversed. The un-tilted state resides at the edge of the spectrum. Increasing θ moves the state toward the central, high-dimension sectors, accelerating relaxation. Thus, QME is present.
- Stark-MBL Regime: Relaxation is governed by dephasing in the effective diagonal basis, and the existence of QME is determined by the ordering of the steady-state values.
  – As derived in Equation (23), the steady-state plateau $\Delta S_{A}^{\left(2\right)}\left(\infty \right)$ scales with $a_{\theta }^{N-2N_{A}}$. Since $a_{\theta }\;=\;\left(1+\cos^{2}\theta \right)/2$ is a monotonically decreasing function of θ, a larger initial tilt θ (higher initial asymmetry) leads to a lower steady-state asymmetry.
  – This inversion of ordering, high initial value leading to low final value, mathematically guarantees that the EA evolution curves for different θ must cross at intermediate times. Consequently, QME becomes universal in the Stark-MBL regime, appearing for both TNS and TFS regardless of their ergodic behavior.

Finally, we estimate the scaling of the Mpemba time $t_{M}$. In the strong Stark limit (F≫J), restoring symmetry within a subsystem of size $N_{A}$ requires quantum fluctuations that couple different local charge sectors. In the effective Hamiltonian picture, such charge reconfiguration is an off-resonant process. Changing the charge configuration across $N_{A}$ sites requires virtually occupying intermediate states up the Stark ladder. According to high-order perturbation theory, the effective matrix element for a process spanning $N_{A}$ sites scales as $J_{\mathrm{eff}}∼J{\left(J/F\right)}^{N_{A}-1}$. The timescale for symmetry restoration, and consequently the timescale on which the QME occurs, is inversely proportional to this rate:

(24) $$t_{M}\propto \frac{1}{J_{\mathrm{eff}}}∼\frac{1}{J}{\left(\frac{F}{J}\right)}^{N_{A}-1}.$$

**Figure 3:**
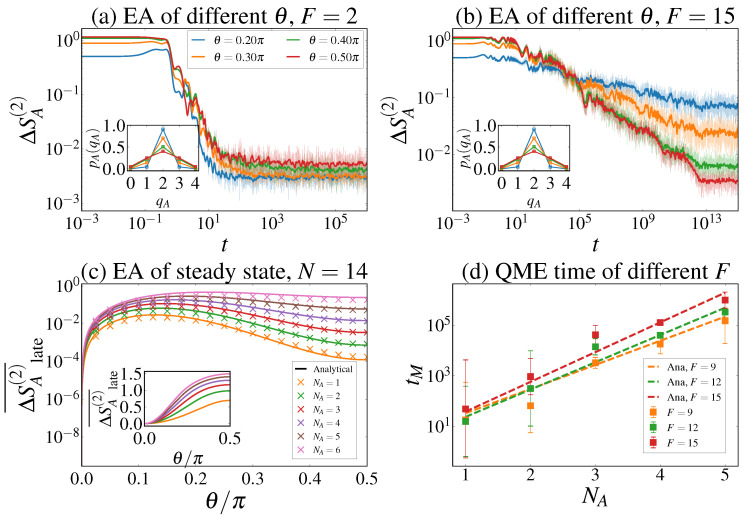
(**a**,**b**) EA dynamics with N=14 and $N_{A}=3$. The initial state is chosen as TNS. The numerical curves have been smoothed by convolution with a Gaussian, $\omega \left(n\right)=e^{-{\left(n/\sigma \right)}^{2}}$, with σ=5. The shaded curves represent the original numerical data before smoothing. Inset in (**a**,**b**): Charge-sector weights $p_{A}\left(q_{A}\right)$ with N=14 and $N_{A}=4$, for the initial TNS. (**c**) Steady-state EA for $F=15>F_{c}$ and different subsystem sizes $N_{A}$, with the initial state prepared as the TNS. Discrete points show the steady-state EA averaged over 2000 samples, while solid lines denote the analytical steady-state values. Inset in (**c**): analytical initial EA at t=0. (**d**) QME time $t_{M}$ as a function of subsystem size $N_{A}$ for different *F* at N=14, starting from the initial TNS. Discrete symbols (with error bars) show the numerically extracted $t_{M}$, while dashed lines denote the theoretical scaling curves obtained by fitting Equation (24) to the numerical data.

**Figure 4:**
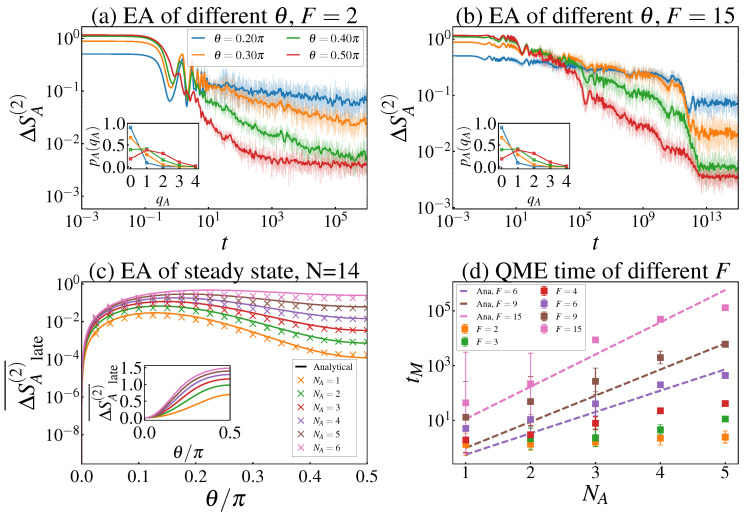
(**a**,**b**) EA dynamics with N=14 and $N_{A}=3$. The initial state is chosen as TFS. The numerical curves have been smoothed by convolution with a Gaussian, $\omega \left(n\right)=e^{-{\left(n/\sigma \right)}^{2}}$, with σ=5. The shaded curves represent the original numerical data before smoothing. Inset in (**a**,**b**): Charge-sector weights $p_{A}\left(q_{A}\right)$ with N=14 and $N_{A}=4$, for the initial TFS. (**c**) Steady-state EA versus θ for $F=15>F_{c}$ and different subsystem sizes $N_{A}$, with the initial state prepared as the TFS. Discrete points show the steady-state EA averaged over late-time 2000 samples, while solid lines denote the analytical steady-state values. Inset in (**c**): analytical initial EA at *t* = 0. (**d**) QME time *t_M_* as a function of subsystem size *N_A_* for different *F* at *N* = 14, starting from the initial TFS. Discrete symbols (with error bars) show the numerically extracted *t_M_*, while dashed lines denote the theoretical scaling curves obtained by fitting Equation (24) to the numerical data.

This prediction implies that the Mpemba time grows exponentially with the subsystem size $N_{A}$ and follows a power-law dependence on the Stark field strength *F*. This confirms that the phenomenon is controlled by the localization length of the system, consistent with the numerical observations in Figure 3b and Figure 4d.

### 3.2. Numerical Results

#### 3.2.1. Stark Many-Body Localized Diagnostics

**Figure 2:**
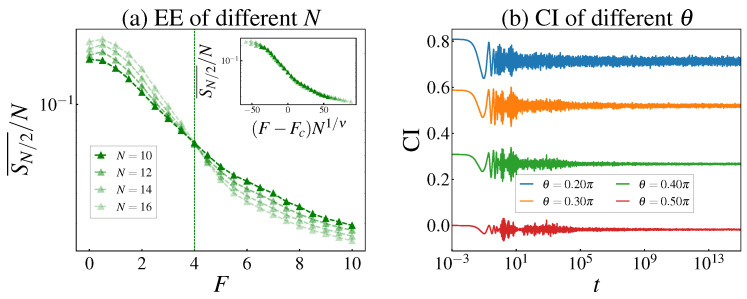
(**a**) The half-chain von Neumann entropy density $\bar{S_{N/2}}/N$ with different *N*. Inset in (**a**): finite-site scaling collapse of $\bar{S_{N/2}}/N$ as a function of $\left(F-F_{c}\right)N^{1/\nu }$, with the critical point $F_{c}≃4.0$. (**b**) Charge imbalance dynamics with N=14 and $F=15>F_{c}$. The initial state is chosen as TNS.

We first establish the parameter regime in which the tilted XXZ chain, described by the Hamiltonian in Equation (1), enters the Stark-MBL phase. To obtain reliable information on the energy spectrum and eigenstates, we numerically solve the static and dynamical properties of the system using the Exact Diagonalization method. Figure 2a shows the half-chain von Neumann entropy density $\bar{S_{N/2}}/N$ as a function of the Stark field *F* for several system sizes *N*. In the small-*F* regime, $\bar{S_{N/2}}/N$ remains finite and exhibits only a mild size dependence, consistent with the volume-law behavior of the ergodic phase. As *F* increases, $\bar{S_{N/2}}/N$ decreases substantially and drifts downward with increasing *N*, indicating a crossover toward the area-law entanglement characteristic of Stark-MBL. The inset of Figure 2a demonstrates a finite-size scaling collapse as a function of $\left(F-F_{c}\right)N^{1/\nu }$, yielding a critical field $F_{c}≃4.0$ (indicated by the vertical green dotted line). Based on this result, we use $F=15>F_{c}$ as a representative Stark-MBL point in our subsequent analysis.

To further obtain dynamical evidence of non-ergodicity, we examine the charge imbalance CI(t) starting from the initial TNS. Figure 2b plots the dynamics of CI(t) at N=14 and F=15 for several tilt angles θ. After an early-time transient, CI(t) rapidly approaches a non-zero long-time plateau. Crucially, the plateau value remains strongly dependent on the initial angle θ, preserving the ordering of the initial conditions over extremely long time scales (up to $t∼10^{15}$). This persistent memory of the initial staggered pattern provides direct dynamical evidence for the non-ergodicity protected by the Stark-MBL phase.

#### 3.2.2. EA Dynamics and the Emergence of QME

Having established the phase diagram, we turn to the core objective: investigating the dynamics of the Rényi-2 EA $\Delta S_{A}^{\left(2\right)}\left(t\right)$ and the resulting QME. To explore how the initial state distribution affects the symmetry restoration process, we first analyze the microscopic composition of the initial states by calculating the charge-sector weights $p_{A}\left(q_{A}\right)=\mathrm{Tr}\left(\Pi_{q_{A}}\rho_{A}\right)$, where $\Pi_{q_{A}}$ is the projection operator onto the eigenspace of the local charge operator $Q_{A}=\sum_{j\in A}S_{j}^{z}$ with eigenvalue $q_{A}$. To further characterize the spatial memory of the initial configurations, we calculate the charge imbalance (CI), which for our spin-$\frac{1}{2}$ system is defined as

(25) $$\mathrm{CI}\left(t\right)=\frac{1}{N}\sum_{i=1}^{N}{\left(-1\right)}^{i}\langle S_{i}^{z}\left(t\right)\rangle .$$

**Figure A1:**
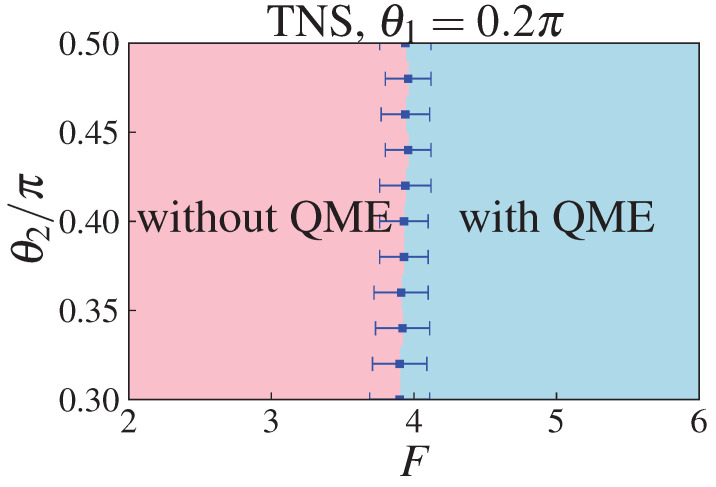
“Phase diagram” of the presence or absence of QME in the $\left(F,\theta_{2}\right)$ plane at fixed $\theta_{1}=0.2\pi$ for the initial TNS. Light-blue (pink) indicates parameter sets with (without) QME. Blue error bars mark the uncertainty of the crossover boundary within our finite-size numerics. Here, N=12 and $N_{A}=3$.

Figure 3a,b show the EA dynamics for the TNS at F=2 and F=15, respectively. At the ergodic point F=2 [Figure 3a], while larger θ corresponds to a larger initial EA, the ordering of the curves does not invert during relaxation, and the QME is absent. The inset shows that $p_{A}\left(q_{A}\right)$ for the TNS is nearly insensitive to θ. However, deep in the Stark-MBL regime (F=15), the EA curves exhibit clear signals, showing the emergence of the QME. A broader scan in the $\left(F,\theta_{2}\right)$ plane for the TNS is provided in Appendix A; see Figure A1, which further supports the absence of QME at small *F* and its emergence beyond a characteristic field scale. In contrast, for the TFS at F=2 [Figure 4a], the QME is already observable, which is highly consistent with the microscopic feature shown in its inset where $p_{A}\left(q_{A}\right)$ significantly shifts toward the central sectors as θ increases.

Then, to verify the accuracy of our aforementioned analytical predictions, we perform a quantitative comparison between the long-time numerical results and the analytical plateau values. In Figure 3c (TNS) and Figure 4c (TFS), we plot the steady-state EA $\Delta S_{A}^{\left(2\right)}\left(t_{\mathrm{ss}}\right)$ versus θ/π for F=15. The discrete symbols represent the numerical results averaged over 2000 samples, while the solid lines denote the analytical values. The results show that the numerical simulations and analytical predictions are in excellent agreement across all subsystem sizes $N_{A}$. This provides strong evidence that our analytical framework quantitatively reproduces the long-time dephasing behavior of Stark-MBL systems under symmetry constraints.

#### 3.2.3. Scaling Characteristics of the Mpemba Time

Finally, we quantify the characteristic timescale of the QME. We define the Mpemba time $t_{M}$ (corresponding to the first intersection of the EA dynamical envelopes) by extracting the first point of the EA curves and fitting the numerical results using the analytically derived scaling relation Equation (24). As shown in Figure 3d and Figure 4d, the fitting curves (dashed lines) perfectly capture the exponential growth of $t_{M}$ with respect to both subsystem size $N_{A}$ and field strength *F*. This not only further validates the theoretical predictions but also reveals that, in the Stark-MBL environment, the QME timescale is dominated by suppressed high-order operator dynamics, where the effective transition rate $J_{\mathrm{eff}}$ decays as ${\left(J/F\right)}^{N_{A}-1}$.

## 4. Conclusions

To sum up, in this work, we have provided a comprehensive analysis of symmetry restoration and the QME within the context of Stark-MBL. By investigating the dynamics of the Rényi-2 EA in a tilted XXZ chain, we found that the Stark-induced breaking of ergodicity leads to a robust and universal Mpemba effect, distinct from its behavior in thermalizing systems.

Our central finding is that, while the occurrence of the QME in the ergodic phase depends on the specific choice of the initial state (e.g., present for TFS but absent for TNS), the Stark-MBL regime imposes a universal inversion of relaxation hierarchies. Through an analytical operator-string expansion, we demonstrated that this universality stems from the structure of the steady-state EA. In the strong-tilt limit, the effective Hilbert space dimension accessible to local observables is strictly constrained by the Stark potential, leading to a “hotter-is-faster” relaxation scenario where states with larger initial asymmetry ultimately approach a more symmetric steady state. Furthermore, we established that the timescale of this anomalous relaxation, the Mpemba time $t_{M}$, is governed by high-order off-resonant processes. We derived a scaling law showing that $t_{M}$ grows exponentially with the subsystem size and follows a power-law dependence on the field strength. This prediction is fully supported by our numerical simulations, which confirm that symmetry restoration in Stark-MBL is driven by slow, perturbative quantum fluctuations rather than by diffusive transport. Our results highlight the unique potential of Stark-MBL systems as tunable platforms for exploring non-equilibrium thermodynamics and symmetry dynamics. Unlike disorder-driven MBL, the Stark potential offers a clean, continuously adjustable control parameter to engineer relaxation pathways. This suggests promising avenues for observing the QME in present-day quantum simulators, such as cold atoms [70,71,72,73,74,75,76] in optical lattices or trapped ions [77], where linear potentials can be implemented with high precision.

Finally, our study opens up several specific directions for future theoretical investigation. While we focused on closed quantum systems, examining the stability of the Mpemba effect under dissipative or non-Hermitian dynamics [78,79,80,81,82,83,84,85,86,87,88,89,90,91,92] would be crucial for understanding its fate in realistic open environments. Additionally, extending the analysis to the framework of nonstabilizerness [55,93,94,95,96,97,98,99,100,101,102,103,104,105,106,107,108,109,110,111,112,113,114,115,116,117,118,119,120,121,122,123,124,125,126,127,128,129,130,131,132,133,134,135,136,137,138,139,140,141,142,143,144,145,146,147] offers a fresh perspective. Utilizing metrics such as the stabilizer Rényi entropy as dynamical probes could quantify the complexity of the relaxation process, potentially revealing a rich interplay between symmetry restoration, localization, and the scrambling of quantum magic.

## Data Availability

The data sets generated and/or analyzed during the current study are available from the corresponding authors upon reasonable request.

## Appendix A. Additional Numerical Results

To further clarify where the QME occurs in parameter space, we provide a “Phase diagram” of the presence or absence of QME in the $\left(F,\theta_{2}\right)$ plane for the TNS at fixed $\theta_{1}=0.2\pi$ (Figure A1), obtained from a systematic survey of the EA dynamics.

We map out a “Phase diagram” of the presence or absence of QME in the $\left(F,\theta_{2}\right)$ plane at fixed $\theta_{1}=0.2\pi$ for the initial TNS (Figure A1) with N=12 and $N_{A}=3$. For each pair $\left(F,\theta_{2}\right)$, we compute the EA dynamics following the same procedure as in the main text and inspect the evolution of $\Delta S_{A}^{\left(2\right)}\left(t\right)$. After smoothing out the fast Stark-induced oscillations, we determine for each $\left(F,\theta_{2}\right)$ whether QME is present according to the criterion defined in the main text, and label the point as “with QME” or “without QME” accordingly. Near the crossover, the can be weak or ambiguous within finite-size numerics, and the blue error bars indicate the corresponding uncertainty in locating the approximate boundary.

Overall, the QME for the TNS is absent at small *F* but becomes clearly visible beyond a characteristic scale around F≃4, consistent with the main text, where no QME is found at small *F*, whereas a clear QME is observed at large *F*.
